# Prognostic value and model construction of preoperative inflammatory markers in patients with metastatic renal cell carcinoma

**DOI:** 10.1186/s12957-023-03110-w

**Published:** 2023-07-21

**Authors:** Jichen Wang, Jiali Ye, Xupeng Zhao, Xiubin Li, Xin Ma

**Affiliations:** 1 Senior Department of Urology, the Third Medical Center of PLA General Hospital, Beijing, China; 2 Medical School of Chinese PLA, Beijing, China; 3 School of Medicine, Nankai University, Tianjin, China

**Keywords:** Inflammatory biomarkers, Metastatic renal cell carcinoma, Overall survival, A prognostic model

## Abstract

**Background:**

Inflammation is considered to be one of the driving factors of cancer, and chronic inflammation plays a crucial role in tumor growth and metastasis. The aim of this study was to examine the predictive value of preoperative inflammatory biomarkers for overall survival (OS) in patients with metastatic renal cell carcinoma (mRCC), including preoperative neutrophil-to-lymphocyte ratio (NLR), lymphocyte-to-monocyte ratio (LMR), and aspartate aminotransferase-to-lymphocyte ratio (ALR), a novel inflammatory biomarker.

**Method:**

This study included 198 patients with mRCC from a single center from 2006 to 2022. The optimal cut-off levels for the three biomarkers were derived using the receiver operating characteristic curve (ROC). Cox univariate and multivariate analyses were used to assess independent prognostic inflammatory biomarkers. Finally, independent prognostic inflammatory biomarkers were incorporated into the prognostic model to establish a nomogram to predict the postoperative survival of patients with mRCC.

**Result:**

The area under the ROC curve for NLR, LMR, and ALR, respectively, is 0.71 (CI: 0.635–0.784), 0.68 (CI: 0.604–0.755), and 0.75 (CI: 0.680–0.819). The optimal LMR, NLR, and ALR cut-off levels as evaluated by the ROC curve were 3.836, 3.106, and 68.056, respectively. Patients with NLR and ALR higher than the cut-off level and LMR lower than the cut-off level had a significant relationship with OS. Multivariate analysis revealed that tumor necrosis, lower LMR, and higher ALR were independent risk factors for OS. In addition, a nomogram that includes independent prognostic inflammatory biomarkers can accurately predict the OS in patients with mRCC.

**Conclusion:**

ALR and LMR are independent risk factors for the prognosis of individuals with mRCC. By monitoring ALR and LMR postoperatively, the prognosis of patients with mRCC can be better evaluated.

## Background

Renal tumors are the second most common urological malignant tumor following bladder cancer [1], with renal cell carcinoma accounting for 90% [2] of all cases. The most prevalent subtype of renal cell carcinoma is clear cell carcinoma, which has a high risk of metastasis and recurrence [3]. Research has shown that individuals with metastasis account for approximately 30% of those diagnosed with renal cell carcinoma [4]. MRCC is a heterogeneous disease that is highly resistant to chemotherapy. Although treatment of mRCC has improved considerably in the past decade [5] and there are numerous therapeutic options available, it did not result in a greater 5-year survival rate of patients, which was only about 10% [6]. Therefore, it is of great importance to explore the relationship between preoperative indicators and the high risk of mRCC. This is crucial in the treatment, intervention, and follow-up of patients.

Several studies have demonstrated some risk factors for OS in patients with mRCC, such as BMI, hyponatremia, nephrectomy, baseline hemoglobin, baseline lactate dehydrogenase (LDH), and so on [7–9]. More and more evidence suggests that inflammation promotes tumors and is even closely related to tumor metastasis and recurrence [10]. With a better understanding of the tumor microenvironment and improved perioperative diagnosis and treatment capabilities in recent years, some inflammatory indicators have been proposed to predict the prognosis of various cancers. For example, the neutrophil-to-lymphocyte ratio (NLR) and lymphocyte-to-monocyte ratio (LMR) have been reported as prognostic biomarkers in lung, colorectal, gastric, esophagus, hepatocellular carcinoma, and kidney cancers [11–16]. Aspartate aminotransferase (AST) is a kind of aminotransferase that is found in multiple organs such as the liver, heart, skeletal muscle, and kidney. Studies have proved that AST and ALT can effectively predict the prognosis of patients with hepatocellular carcinoma, renal cell carcinoma, and breast cancer [17, 18]. The aspartate aminotransferase to lymphocyte ratio (ALR), a novel inflammatory biomarker, has only been used in hepatocellular carcinoma and has yet to be proven prognostic [19]. However, inflammatory biomarkers, especially inflammatory index combination, have not been extensively studied in mRCC.

As a result, the objective of this study was to demonstrate the prognostic relevance of NLR and LMR for mRCC patients, as well as to evaluate the potential prognostic impact of ALR on mRCC patients and to explore its relationship with OS. Finally, to assess the value of inflammatory biomarkers in the prognosis of mRCC patients. ALR and other independent prognostic factors were employed to construct a nomogram, by using this nomogram, we can accurately predict the survival status of mRCC patients 1 and 2 years after surgery, which can provide urologists with appropriate ideas for follow-up diagnosis and treatment. In addition, compared with other complex and expensive detection indicators, this new biomarker can also be used as a convenient and inexpensive indicator to assist in the prognosis assessment of mRCC patients.

## Method

### Patient

The medical records of 212 patients with mRCC from a single center between 2006 and 2021 were retrospectively collected, and subsequent retrospective analyses were conducted. Patients with incomplete data were excluded (*n* = 14), yielding 198 patients in the final cohort. The inclusion criteria are as follows: 1) Patients with renal cell carcinoma who have clear evidence of metastasis to other sites before surgery. 2) Unilateral kidney cancer. 3) Negative surgical margin. 4) No severe inflammation, infection, high fever, blood disease, or kidney rupture. 5) Informed consent was obtained from all eligible patients, and all medical records were approved by the Ethics Committee of our hospital.

Collect routine examination data after admission: including demographic data and medical history, hematology, and laboratory data 1 week before surgery, AST, surgical method, metastatic organs, postoperative pathological parameters, and postoperative medication. Surgical methods include open surgery and minimally invasive surgery, including robotic and laparoscopic nephrectomy. Postoperative pathological parameters comprised pathological cell type, pathologic Fuhrman grading, and TNM grade based on the International Union Against Cancer (UICC). Fuhrman grading is a histopathological grading system used to evaluate the aggressiveness and prognosis of RCC. It classifies RCC into four grades (grades 1 to 4) based on the characteristics of tumor cell nuclei. Higher Fuhrman grades indicate more aggressive and less differentiated tumor cells, correlating with poorer prognosis and increased risk of disease progression. MRCC patients are more likely to relapse after resection of the tumor only through surgery, while postoperative medication therapy can reduce the probability of recurrence. Postoperative medications are categorized as programmed cell death protein 1 (PD1) or thymidine kinase 1 (TK1) drugs. The calculation formulas of NLR, LMR, and ALR are as follows:

$$\mathrm{NLR }=\mathrm{ neutrophil count}/\mathrm{lymphocyte count ratio};\mathrm{ LMR }=\mathrm{ lymphocyte count}/\mathrm{monocyte count ratio};\mathrm{ ALR }=\mathrm{ aspartate aminotransferase}/\mathrm{lymphocyte count ratio}.$$

### Follow up

Following postoperative discharge, the patient underwent regular outpatient follow-up checks every three weeks for the first two years, every two months for the third to fifth years, and then every year thereafter. The inspection contents include a blood routine examination, blood biochemical examination, and abdominal ultrasonography. The primary study outcome of this study was OS, which was defined as the time from the end of surgery to death or the final follow-up. The latest date for a follow-up date is December 31, 2021.

### Statistical analysis

Most statistical studies in this project were analyzed using R software 3.6.3 (R Foundation for Statistical Computing, Vienna, Austria). Continuous variables were expressed as the median (interquartile range, (IQR)), and categorical variables were expressed as counts and percentages. The optimal cut-off levels of the inflammatory index were identified using the Youden index and computed using the Receiver operating characteristic (ROC) curves, and the area under the curve(AUC) significance test was performed using DeLong's test. Pearson's chi-square test or Fisher's exact test was used to determine the correlation between NLR, LMR, and ALR and each clinical variable. The inflammatory biomarkers were divided into two groups according to the optimal cutoff levels, survival curves were drawn utilizing Kaplan–Meier survival analysis, and the log-rank test was applied for significance comparison. The three inflammatory indicators, as well as other important clinicopathological parameters (gender, age, hemoglobin, postoperative medication, tumor site, number of metastatic sites, histology, microvascular invasion, tumor size, nephrectomy, Fuhrman grade, T stage, N stage, tumor necrosis), were subjected to univariate Cox regression analysis, and the univariate analysis variables with *P* ≤ 0.05 were included in the multivariate Cox regression analysis to clarify the independent prognostic factors of OS and used (Concordance, C- index) for consistency check analysis. The nomogram was developed collaboratively integrating independent prognostic factors, and its predictive accuracy was evaluated. In this study, *P* ≤ 0.05 was defined as a statistical difference.

## Result

### Baseline characteristics of patients

Table 1 shows the clinical and pathological characteristics of the patients. This work comprised 198 patients, including 154 (77.8%) males and 44 (22.2%) females. Eighty-two (41.4%) patients had passed away and 116 (58.6%) were still living by the last follow-up.

**Table 1:** Baseline characteristics of patients

Characteristics	overall
Gender, n (%)	
Male	154 (77.8%)
Female	44 (22.2%)
Age, median (IQR)	57 (49, 63)
Fuhrman grade, n (%)	
G1 + G2	108 (54.5%)
G3 + G4	90 (45.5%)
Postoperative medication, n (%)	
Absent	35 (17.7%)
TK1	25 (12.6%)
PD1	138 (69.7%)
Tumor site, n (%)	
Left	120 (60.6%)
Right	78 (39.4%)
Number of metastatic sites, n (%)	
< 2	151 (76.3%)
≥ 2	47 (23.7%)
Histology, n (%)	
Clear cell	176 (88.9%)
Non–clear cell	22 (11.1%)
Microvascular invasion, n (%)	
Present	47 (23.7%)
Absent	151 (76.3%)
Tumor size (cm), n (%)	
> 7	116 (58.6%)
≤ 7	82 (41.4%)
Nephrectomy, n (%)	
Minimally invasive	176 (88.9%)
Open	22 (11.1%)
T stage, n (%)	
T1 + T2	54 (27.3%)
T3 + T4	144 (72.7%)
N stage, n (%)	
N0	147 (74.2%)
N1	51 (25.8%)
Tumor necrosis, n (%)	
Present	98 (49.5%)
Absent	100 (50.5%)

**Fig. 1:**
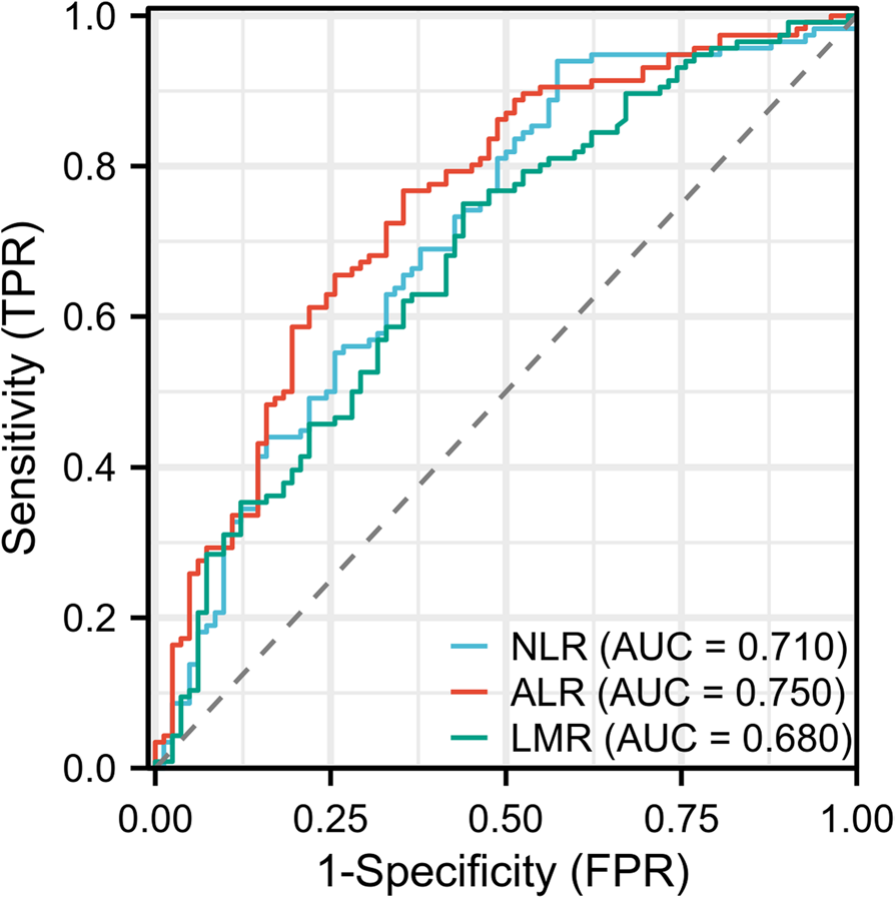
Receiver operating characteristic analysis of NLR, ALR, and LMR. Abbreviations: NLR stands for neutrophil-to-lymphocyte ratio; ALR stands for aspartate aminotransferase-to-lymphocyte ratio; LMR stands for lymphocyte-to-monocyte ratio; and AUC stands for the area under the curve

Figure 1 shows the ROC curves of ALR, NLR, and LMR, with AUC areas of 0.75 (CI: 0.680–0.819), 0.71 (CI: 0.635–0.784), and 0.68 (CI: 0.604–0.755), respectively. According to the ROC curve, the best cut-off levels for the three indicators are calculated as 68.056, 3.836, and 3.106, respectively, while the Youden indices are 0.414, 0.366, and 0.311 respectively. To facilitate the analysis of the relationship between the three indicators and the disease, each of the three indicators was divided into two groups: high ALR (> 68.056) group and low ALR (≤ 68.056) group, high NLR (> 3.836) group and low NLR (≤ 3.836) group, and high LMR (> 3.106) group and low LMR (≤ 3.106) group.

Table 2 shows the relationship between clinicopathological parameters and three inflammatory biomarkers. There were 80 (40.4%) cases in the high ALR group and 118 (59.6%) cases in the low ALR group, 42 (21.2%) cases in the high NLR group, and 156 (78.8%) cases in the low NLR group, and 123 (62.1%) cases in the low LMR group and 75 (37.9%) cases in the high LMR group. Overall, patients with higher ALR exhibited higher T stages (*P* ≤ 0.05), while patients with higher NLR had a higher risk of tumor necrosis (*P* ≤ 0.05). However, there was no statistical significance between LMR and gender, age, Fuhrman grade, postoperative medication, tumor site, number of metastatic sites, histology, microvascular invasion, tumor size, nephrectomy, T stage, N stage, and tumor necrosis (*p* > 0.05).

**Table 2:** Baseline parameters of the study

Parameters^b^		ALR > 68.056	ALR ≤ 68.056	*P* value	NLR > 3.836	NLR ≤ 3.836	*P* value	LMR > 3.106	LMR ≤ 3.106	*P* value^a^
All patients		80	118		42	156		75	123	
Gender, n (%)	Male	60 (30.3%)	94 (47.5%)	0.549	29 (14.6%)	125 (63.1%)	0.185	59 (29.8%)	95 (48%)	0.953
	Female	20 (10.1%)	24 (12.1%)		13 (6.6%)	31 (15.7%)		16 (8.1%)	28 (14.1%)	
Age n (%)	> 60	31 (15.7%)	42 (21.2%)	0.763	16 (8.1%)	57 (28.8%)	0.996	30 (15.2%)	43 (21.7%)	0.575
	≤ 60	49 (24.7%)	76 (38.4%)		26 (13.1%)	99 (50%)		45 (22.7%)	80 (40.4%)	
Fuhrman grade, n (%)	G1 + G2	41 (20.7%)	67 (33.8%)	0.534	21 (10.6%)	87 (43.9%)	0.623	36 (18.2%)	72 (36.4%)	0.195
	G3 + G4	39 (19.7%)	51 (25.8%)		21 (10.6%)	69 (34.8%)		39 (19.7%)	51 (25.8%)	
Targeted therapy, n (%)	Absent	18 (9.1%)	17 (8.6%)	0.275	5 (2.5%)	30 (15.2%)	0.535	15 (7.6%)	20 (10.1%)	0.796
	TK1	11 (5.6%)	14 (7.1%)		6 (3%)	19 (9.6%)		9 (4.5%)	16 (8.1%)	
	PD1	51 (25.8%)	87 (43.9%)		31 (15.7%)	107 (54%)		51 (25.8%)	87 (43.9%)	
Tumor site, n (%)	Left	51 (25.8%)	69 (34.8%)	0.550	28 (14.1%)	92 (46.5%)	0.467	45 (22.7%)	75 (37.9%)	1.000
	Right	29 (14.6%)	49 (24.7%)		14 (7.1%)	64 (32.3%)		30 (15.2%)	48 (24.2%)	
Number of metastatic sites, n (%)	< 2	55 (27.8%)	96 (48.5%)	0.061	30 (15.2%)	121 (61.1%)	0.532	56 (28.3%)	95 (48%)	0.810
	≥ 2	25 (12.6%)	22 (11.1%)		12 (6.1%)	35 (17.7%)		19 (9.6%)	28 (14.1%)	
Histology, n (%)	Clear cell	70 (35.4%)	106 (53.5%)	0.778	36 (18.2%)	140 (70.7%)	0.422	69 (34.8%)	107 (54%)	0.393
	Non–clear cell	10 (5.1%)	12 (6.1%)		6 (3%)	16 (8.1%)		6 (3%)	16 (8.1%)	
Microvascular invasion, n (%)	Present	23 (11.6%)	24 (12.1%)	0.232	14 (7.1%)	33 (16.7%)	0.149	21 (10.6%)	26 (13.1%)	0.353
	Absent	57 (28.8%)	94 (47.5%)		28 (14.1%)	123 (62.1%)		54 (27.3%)	97 (49%)	
Tumor size (cm), n (%)	> 7	41 (20.7%)	75 (37.9%)	0.114	21 (10.6%)	95 (48%)	0.273	39 (19.7%)	77 (38.9%)	0.187
	≤ 7	39 (19.7%)	43 (21.7%)		21 (10.6%)	61 (30.8%)		36 (18.2%)	46 (23.2%)	
Nephrectomy, n (%)	Minimally invasive	73 (36.9%)	103 (52%)	0.522	39 (19.7%)	137 (69.2%)	0.579	68 (34.3%)	108 (54.5%)	0.698
	Open	7 (3.5%)	15 (7.6%)		3 (1.5%)	19 (9.6%)		7 (3.5%)	15 (7.6%)	
T stage, n (%)	T1 + T2	13 (6.6%)	41 (20.7%)	**0.007**	6 (3%)	48 (24.2%)	0.053	19 (9.6%)	35 (17.7%)	0.754
	T3 + T4	67 (33.8%)	77 (38.9%)		36 (18.2%)	108 (54.5%)		56 (28.3%)	88 (44.4%)	
N stage, n (%)	N0	57 (28.8%)	90 (45.5%)	0.531	29 (14.6%)	118 (59.6%)	0.504	50 (25.3%)	97 (49%)	0.083
	N1	23 (11.6%)	28 (14.1%)		13 (6.6%)	38 (19.2%)		25 (12.6%)	26 (13.1%)	
Tumor necrosis, n (%)	Present	43 (21.7%)	55 (27.8%)	0.400	27 (13.6%)	71 (35.9%)	**0.047**	43 (21.7%)	55 (27.8%)	0.115
	Absent	37 (18.7%)	63 (31.8%)		15 (7.6%)	85 (42.9%)		32 (16.2%)	68 (34.3%)	

The difference between groups was tested using the Chi-squared test

^a^Statistically significant results were in bold

^b^*Abbreviations*: NLR stands for neutrophil-to-lymphocyte ratio; ALR stands for aspartate aminotransferase-to-lymphocyte ratio; and LMR stands for lymphocyte-to-monocyte ratio

### Associations of ALR, NLR, and LMR with OS

**Fig. 2:**
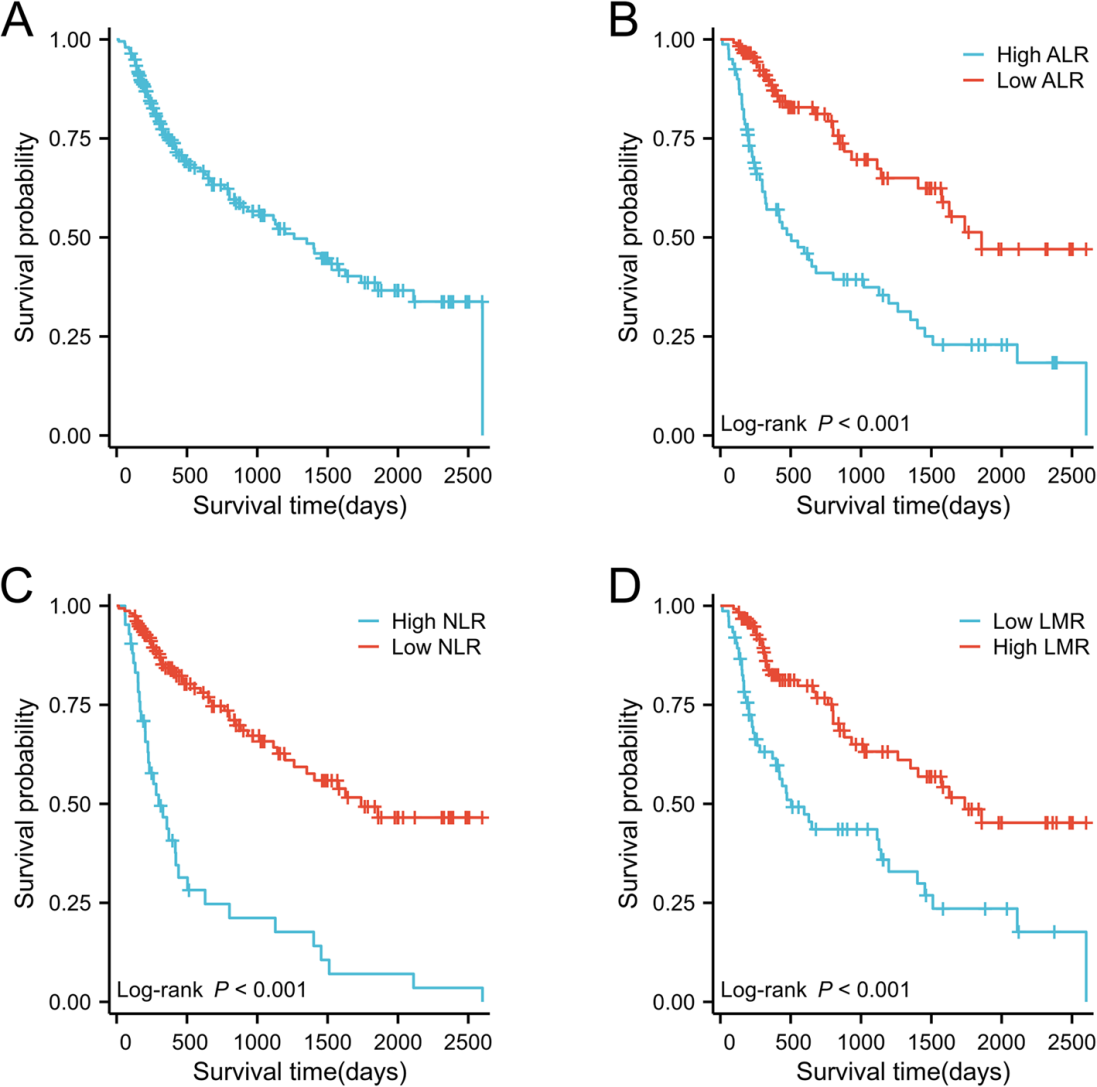
Kaplan–Meier curves for overall survival probability according to NLR, ALR, and LMR. **a** OS of patients with mRCC; **b** Kaplan–Meier curve of OS based on ALR level; **c** Kaplan–Meier curve of OS based on NLR level; **d** Kaplan -Meier curve of OS based on LMR level

As shown in Fig. 2, Kaplan–Meier survival analysis was performed utilizing the optimal cut-off level, and the results showed that patients with low levels of ALR (HR = (0.33 (0.21 − 0.52)) and NLR (HR = 0.23 (0.15 − 0.36)) had a longer OS, whereas patients with high LMR (HR = 2.57 (1.66 − 3.99)) had a shorter OS. The Log-rank test revealed that all three indicators were statistically significant (*P* < 0.001).

### Univariate and multivariate Cox regression survival analysis

Table 3 depicts the relationship between clinicopathological parameters and ALR, NLR, LMR, and OS. The univariate analysis revealed that hemoglobin, histology, nephrectomy, T stage, tumor necrosis, ALR, NLR, and LMR were prognostic factors for OS, whereas other variables showed no statistical difference (*P* > 0.05). This study demonstrated that tumor necrosis (HR, 0.524; 95% CI, 0.328–0.838; *P* = 0.007), LMR (HR, 0.783; 95% CI, 0.650–0.943; *P* = 0.01), and ALR (HR, 1.005; 95% CI, 1.001–1.008; *P* = 0.006) were independent risk factors for OS as we included important characteristics from univariate analysis into multivariate analysis.

**Table 3:** Univariate and multivariate Cox regression survival analysis for the prediction of OS

Characteristics^b^	Total(N)	Univariate analysis		Multivariate analysis	
		Hazard ratio (95% CI)	*P* value^a^	Hazard ratio (95% CI)	*P* value^a^
Gender	198				
Male	154	Reference			
Female	44	1.161 (0.687–1.962)	0.577		
Age	198	1.004 (0.986–1.022)	0.687		
Hemoglobin	198	0.987 (0.978–0.995)	**0.002**	0.991 (0.981–1.001)	0.065
Postoperative medication	198				
Absent	35	Reference			
TK1	25	0.747 (0.365–1.530)	0.425		
PD1	138	0.680 (0.396–1.167)	0.162		
Tumor site	198				
Left	120	Reference			
Right	78	0.951 (0.607–1.490)	0.828		
Number of metastatic sites	198				
< 2	151	Reference			
≥ 2	47	1.123 (0.683–1.848)	0.648		
Histology	198				
Clear cell	176	Reference			
Non–clear cell	22	2.235 (1.216–4.108)	**0.010**	1.820 (0.964–3.436)	0.065
Microvascular invasion	198				
Present	47	Reference			
Absent	151	0.823 (0.491–1.379)	0.459		
Tumor size (cm)	198				
> 7	116	Reference			
≤ 7	82	1.379 (0.891–2.133)	0.149		
Nephrectomy	198				
Minimally invasive	176	Reference			
Open	22	0.321 (0.117–0.879)	**0.027**	0.397 (0.140–1.126)	0.082
Fuhrman grade	198				
G1 + G2	108	Reference			
G3 + G4	90	1.407 (0.909–2.178)	0.126		
T stage	198				
T1 + T2	54	Reference			
T3 + T4	144	1.917 (1.094–3.358)	**0.023**	1.263 (0.702–2.271)	0.436
N stage	198				
N0	147	Reference			
N1	51	1.021 (0.610–1.708)	0.937		
Tumor necrosis	198				
Present	98	Reference			
Absent	100	0.542 (0.348–0.844)	**0.007**	0.524 (0.328–0.838)	**0.007**
LMR	198	0.736 (0.625–0.867)	** < 0.001**	0.783 (0.650–0.943)	**0.010**
NLR	198	1.127 (1.049–1.211)	**0.001**	0.948 (0.823–1.091)	0.457
ALR	198	1.007 (1.005–1.010)	** < 0.001**	1.005 (1.001–1.008)	**0.006**

The difference between groups was tested using the Chi-squared test

^a^Statistically significant results were in bold

^b^*Abbreviations*: NLR stands for neutrophil-to-lymphocyte ratio; ALR stands for aspartate aminotransferase-to-lymphocyte ratio; and LMR stands for lymphocyte-to-monocyte ratio

### Prognostic nomogram for OS

**Fig. 3:**
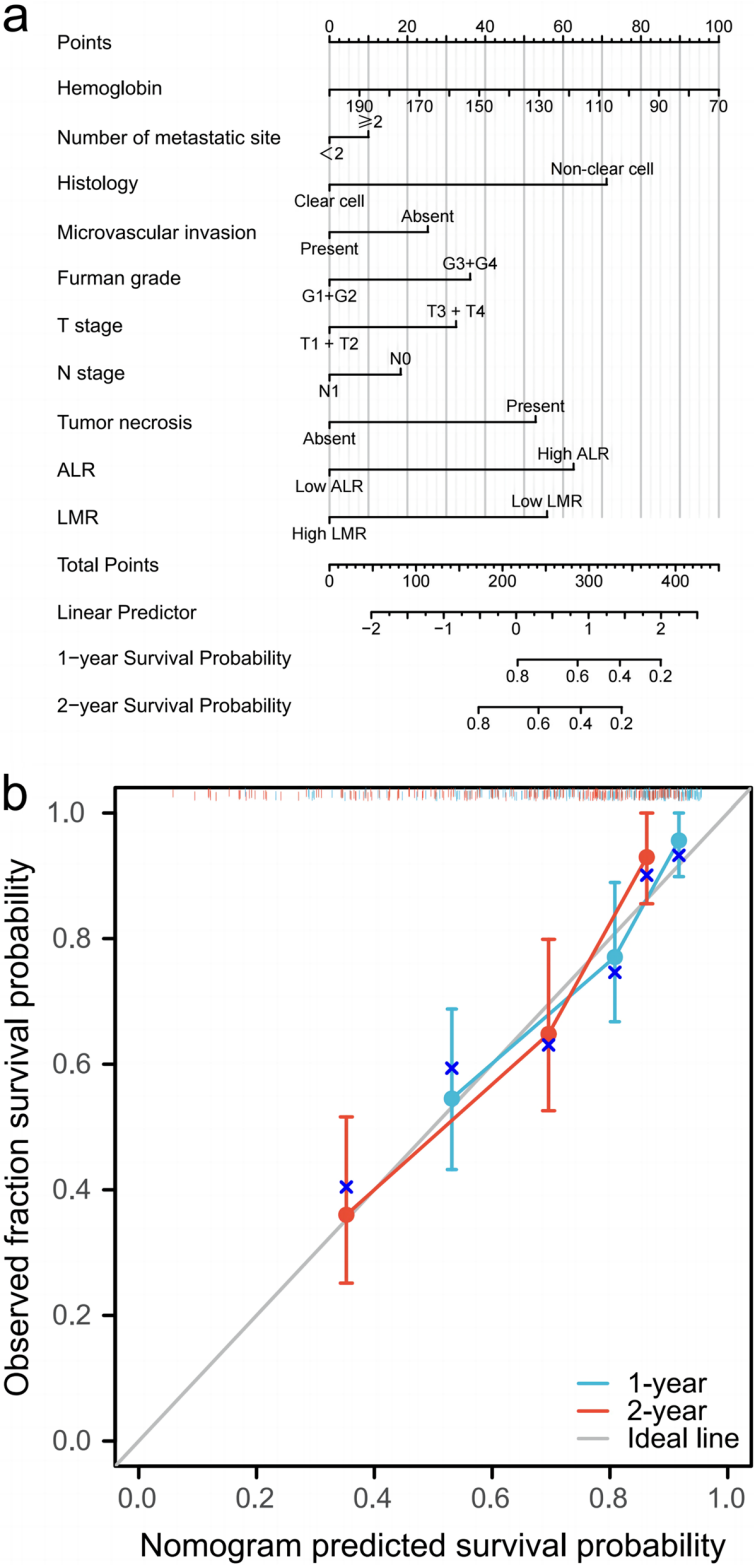
Nomogram for predicting 1- and 2-year OS of mRCC patients after operation. **a** Nomogram for predicting 1- and 2-year OS of mRCC patients after operation. **b** Calibration plot of the nomogram for 1-year and 2-year survival. Each line represents the comparison of the survival situation with the actual situation at each time point, as well as the most ideal line (diagonal: gray); the closer to the diagonal, the better the fit. The vertical line corresponding to the point of each line reflects the confidence interval for that position. The blue cross on each line represents the result of each point after the stratified Kaplan–Meier correction. The vertical line at the top represents the survival probability corresponding to the specific sample (survival distribution). The greater the density, the greater the sample's survival probability within this probability

To render the outcomes of the prediction model more comprehensible, we combined independent risk factors with some clinically important pathological parameters (hemoglobin, number of metastatic sites, histology, microvascular invasion, Fuhrman grade, T stage, N stage, tumor necrosis, LMR, and ALR) in the prognostic nomogram (Fig. 3). The nomogram predicted the 1-year and 2-year survival of patients with mRCC after the operation, and the higher the total point, the worse the prognosis. Following that, we calibrated the constructed nomogram for prognosis. This figure displays the difference between the predicted and the actual probability corresponding to the model after 1 year and 2 years. It can be seen that the survival projected by the nomogram corresponds to the actual scenario, indicating that the forecast fits well (Fig. 3). The C-index of the consistency test was 0.753 (0.727–0.779) (*P* < 0.001).

## Discussion

Patients with renal cell carcinoma account for 90% of people with renal tumors [2]. Localized renal cell carcinoma is generally curable with surgery, while metastatic renal cell carcinoma often has poor treatment effects [20], with a 5-year survival rate of fewer than 10% [6]. Therefore, it is necessary to discover independent risk factors for these patients.

At present, several studies have established that the prognosis of patients with mRCC is related to a variety of factors. For example, Laurence Albiges and others demonstrated in 2016 that there is a close relationship between BMI and the prognosis of patients with mRCC [7]; while ANJeppesen and others showed in 2010 that hyponatremia is an independent prognostic and predictive factor for patients with mRCC [8]. Furthermore, various tumor-related parameters, such as nephrectomy, baseline hemoglobin, baseline lactate dehydrogenase (LDH), and alkaline phosphatase (ALP) [9] have been reported as predictive variables for mRCC survival. Interestingly, previous studies have confirmed that neutrophil count has been incorporated into the International metastatic RCC Database Alliance (IMDC) model, which is one of the most commonly applied prognostic models for stratifying mRCC patients into risk groups [21]. However, as a more sensitive and accurate indicator, inflammatory biomarkers such as ALR were not taken into account at the time. In this study, we will further explore the relationship between inflammatory biomarkers and the prognosis of patients with mRCC so as to find complementing biomarkers.

In this study, 198 mRCC patients' clinicopathological indicators were included. The findings of COX proportional hazards regression proved that LMR and ALR are independent risk factors for OS. NLR was an essential indicator in univariate analysis, but it displayed little statistical value in multivariate analysis. Adding on, patients with higher ALR had higher T stages, while patients with higher NLR were more prone to tumor necrosis. Thus, we integrated the two inflammatory indicators, ALR and LMR, with some conventional clinicopathological indicators to form a nomogram to predict the 1-year and 2-year survival rate of mRCC patients after surgery, which the calibration map matches well.

In recent years, more and more evidence has emerged indicating a close relationship between inflammation and cancer, and the predictive value of two common inflammatory biomarkers (NLR and LMR) in cancer has been confirmed [22–24]. NLR and LMR have been established in studies to have considerable prognostic relevance in individuals with non-metastatic renal cell carcinoma following surgery [25, 26]. In addition, studies have demonstrated the prognostic value of NLR on mRCC patients after surgery [27, 28]. As inflammatory immune cells, neutrophils alter the tumor microenvironment by expressing chemokine receptors CXCR1 and CXCR2 [29]. Moreover, neutrophils release reactive oxygen species (ROS) and proteases, which aid in cancer development [30]. Monocyte-derived macrophages secrete oncogenic factors or respond to cancer-associated cytokines to promote tumorigenesis. Lymphocytes have a prominent influence on the immunological response [31]. T-lymphocytes have the ability to directly eliminate cancerous cells, but B-lymphocytes can produce immunoglobulins to kill and inhibit cancer cells by producing IgM antibodies. A decline in LMR suggests a decrease in lymphocyte count or a relative or absolute increase in monocyte count, reflecting the weakening of the body's anti-tumor function. This is consistent with our hypothesis that a low LMR level corresponds to a poor prognosis.

Aminotransferases, which comprise AST and alanine aminotransferase (ALT), are liver enzymes produced by malignant or non-malignant cells and have been proven to be biomarkers of various malignant tumors such as lung cancer, breast cancer, and pancreatic cancer [32–34]. ALT is a liver-specific index. Considering AST is more widely distributed in the body than ALT [17], exploring the relationship between AST and non-hepatic diseases is more relevant. Although studies have shown that the ratio of the AST/ALT is an important prognostic factor for urinary system tumors [35], there is a lack of studies on the influence of the ratio of ALR on the prognosis of urinary tumors. The influence of liver metastases on the prediction accuracy of ALR indicators can be ruled out since only 12 of the 198 patients in this research had liver metastases.

In basic research, the "Warburg effect" was put forward by Otto Heinrich Warburg. He found that cancer tissue employs glycolysis as the primary route of ATP synthesis even in an aerobic environment and performs a higher aerobic glycolysis rate compared with normal tissue [36]. As a major enzyme in the malate-aspartate shuttle pathway in glycolysis, AST plays an essential part in increasing the aerobic glycolysis and glutamine synthesis of cancer cells. Additionally, about 80% of RCC patients have VHL gene mutations [37], which upregulates the hypoxia-inducible factor (HIF) and produces a pseudo-hypoxic state, which in turn increases glycolysis [38]. All of this has laid a potentially sound theoretical framework for this paper.

This study has identified ALR and LMR as independent prognostic factors for mRCC. This finding deepens our understanding of the tumor inflammatory process and provides novel avenues for treatment monitoring and prognosis assessment in mRCC patients. We hope our study may provide some evidences for helping clinicians to optimize personalized treatment strategies, improve treatment efficacy. Ultimately, these advancements aim to enhance the survival rate and quality of life for individuals with mRCC.

Again, there are limitations in this study. To begin, we only collected data on inflammatory markers and did not collect other important markers such as serum albumin and C-reactive protein. These markers may have an important role in the occurrence and development of mRCC, although further research will be conducted on this aspect later. Second, while a single center is beneficial for controlling the confounding factors affecting prognosis such as surgical procedures, it is also prone to bias. A larger sample and multicenter prospective studies will be conducted in the future to corroborate the findings. Last, this study did not choose progression-free survival as a study outcome for lucubrating, and we will recruit more patients for future research.

## Conclusions

This study included a total of 198 patients with mRCC, and the results demonstrated that NLR, LMR, and a novel inflammatory marker ALR can be used as predictors to evaluate the prognosis of mRCC patients. Although multivariate COX regression analysis confirmed that NLR is not an independent risk factor for mRCC prognosis, it is apparent that NLR is still a significant factor affecting mRCC prognosis. ALR and LMR are inexpensive and convenient to assess as independent prognostic variables. It may be considered to combine them with conventional pathological parameters to enhance the prognosis assessment of mRCC patients.

## Data Availability

The datasets used and/or analysed during the current study are available from the corresponding author on reasonable request.

## Abbreviations

OS: Overall survival
mRCC: Metastatic renal cell carcinoma
NLR: Neutrophil-to-lymphocyte ratio
ALR: Aspartate aminotransferase-to-lymphocyte ratio
LMR: Lymphocyte-to-monocyte ratio
AUC: Area under the curve
ROC: Receiver operating characteristic curve
UICC: International Union Against Cancer
PD1: Programmed cell death protein 1
TK1: Thymidine kinase 1
IQR: Interquartile range
